# Alternative Complement Pathway Inhibition Abrogates Pneumococcal Opsonophagocytosis in Vaccine-Naïve, but Not in Vaccinated Individuals

**DOI:** 10.3389/fimmu.2021.732146

**Published:** 2021-10-11

**Authors:** Lukas Muri, Emma Ispasanie, Anna Schubart, Christine Thorburn, Natasa Zamurovic, Thomas Holbro, Michael Kammüller, Gerd Pluschke

**Affiliations:** ^1^ Molecular Immunology Unit, Swiss Tropical and Public Health Institute, Basel, Switzerland; ^2^ University of Basel, Basel, Switzerland; ^3^ Translational Medicine-Preclinical Safety, Novartis Institutes for Biomedical Research, Basel, Switzerland; ^4^ Novartis Pharma AG, Global Drug Development, London, United Kingdom; ^5^ Novartis Pharma AG, Global Drug Development, Basel, Switzerland

**Keywords:** complement system, *Streptococcus pneumoniae*, opsonophagocytosis, alternative pathway inhibitor, microbial immunology, innate immunity

## Abstract

To assess the relative contribution of opsonisation by antibodies, classical and alternative complement pathways to pneumococcal phagocytosis, we analyzed killing of pneumococci by human blood leukocytes collected from vaccine-naïve and PCV13-vaccinated subjects. With serotype 4 pneumococci as model, two different physiologic opsonophagocytosis assays based on either hirudin-anticoagulated whole blood or on washed cells from EDTA-anticoagulated blood reconstituted with active serum, were compared. Pneumococcal killing was measured in the presence of inhibitors targeting the complement components C3, C5, MASP-2, factor B or factor D. The two assay formats yielded highly consistent and comparable results. They highlighted the importance of alternative complement pathway activation for efficient opsonophagocytic killing in blood of vaccine-naïve subjects. In contrast, alternative complement pathway inhibition did not affect pneumococcal killing in PCV13-vaccinated individuals. Independent of amplification by the alternative pathway, even low capsule-specific antibody concentrations were sufficient to efficiently trigger classical pathway mediated opsonophagocytosis. In heat-inactivated or C3-inhibited serum, high concentrations of capsule-specific antibodies were required to trigger complement-independent opsonophagocytosis. Our findings suggest that treatment with alternative complement pathway inhibitors will increase susceptibility for invasive pneumococcal infection in non-immune subjects, but it will not impede pneumococcal clearance in vaccinated individuals.

## 1 Introduction

The complement system is a complex protein network with essential functions during immunological and inflammatory processes (1). Complement activation induces direct killing of certain pathogens *via* insertion of the pore-forming membrane attack complex (MAC, C5b-9), and opsonizes invading bacteria for subsequent engulfment by phagocytes (1, 2). While MAC insertion into the outer membrane of Gram-negative bacteria like meningococci (*Neisseria meningitidis*) can kill them efficiently within minutes (1–3), Gram-positive bacteria are generally resistant to MAC-induced bacteriolysis, as the terminal complement complex cannot penetrate through the thick peptidoglycan layer of the Gram-positive cell wall (4, 5). The peptidoglycan layer itself, however, might not represent the only resistance mechanism of Gram-positive bacteria to MAC insertion, as recent publications additionally reported the release of specific proteins from Gram-positive bacteria that interfere with MAC assembly (5–7).

*Streptococcus pneumoniae* is a Gram-positive, extracellular, opportunistic pathogen, which may colonize the mucosa of the human upper respiratory tract (8). Although pneumococcal colonization typically manifests as a commensal relationship with its host (8, 9), pneumococci may also cause a range of invasive diseases including otitis media, sepsis, pneumonia and meningitis (8). Pneumococcal clearance from the bloodstream depends mostly on antibody-mediated opsonophagocytosis enhanced by classical complement pathway (CP)-mediated deposition of C3b on the bacterial cell surface with subsequent recognition by phagocyte C3 receptors (10, 11). Data from humans with homozygous C3 deficiencies further highlighted the importance of C3 during pneumococcal infections, as human C3 deficiency is associated with recurrent and life-threatening bacterial infections by encapsulated bacteria such as *S. pneumoniae*, *N. meningitidis* and *Haemophilus influenza* (12, 13). Deficiencies in alternative complement pathway (AP) components also increase susceptibility to bacterial infections. Factor D (fD) and properdin deficiencies are associated with meningococcal and pneumococcal diseases (14–18). Factor B (fB) deficiency has only been reported in two patients so far, which presented with recurring invasive pneumococcal and meningococcal disease (19, 20). The role of the lectin pathway (LP) during pneumococcal infection remains inconclusive (21), with large studies demonstrating no association between human LP deficiencies and the risk of pneumococcal infection (22, 23), despite affecting disease severity (22).

The pneumococcal polysaccharide capsule represents the major virulence determinant and the immunodominant surface structure of *S. pneumoniae* (8). After discovering the immunogenicity of purified pneumococcal polysaccharides, polyvalent pneumococcal polysaccharide vaccines (PPVs) were introduced – initially targeting 2 serotypes and progressing to the development of a 23-valent formulation in the early 1980s (24, 25). This eventually resulted in a coverage of up to 95% of circulating invasive pneumococcal strains depending on geographical location (26). PPVs have been demonstrated to be efficacious in preventing invasive pneumococcal disease in adults (27). However, in the elderly population, vaccine efficacy seems to be reduced, and protection against non-bacteremic pneumonia remains controversial (28–31). Together with the lack of immunogenicity of pure polysaccharide vaccines in children below the age of 2 years, PPVs provide suboptimal clinical efficacy for the two largest risk groups of pneumococcal disease (32).

Because of the poor immunogenicity of the T cell-independent pure polysaccharide vaccines in risk groups together with limited duration of protective antibody titers, pneumococcal conjugate vaccines (PCV) were developed (27, 32). By covalently conjugating capsular polysaccharides to immunogenic carrier proteins, the pneumococcal polysaccharides elicit T cell-dependent immune responses with improved immunological memory, which also reduce nasopharyngeal carriage (8, 32–35). In contrast to the PPV response, which relies on specific splenic B cell subsets that are not fully developed before the age of 2 (36, 37), PCVs elicit protective antibody responses in infants and children below the age of 2 (38). PCV13 was shown to provide significant protection against all vaccine serotypes – but serotype 3 – in young children (39) and also reduced vaccine-type pneumonia and invasive disease in the vaccinated elderly population over a 5-year period (40).

Dysregulation of complement activation causes a number of diseases, including paroxysmal nocturnal hemoglobinuria (PNH), C3 glomerulopathy (C3G) and atypical hemolytic uremic syndrome (aHUS) (41). Current treatment of such diseases includes prevention of the complement membrane attack complex (MAC) with monoclonal antibodies (mAbs) that bind to C5 (41). While MAC formation is involved in uncontrolled lysis of erythrocytes in some of these patients, it is also required for serum bactericidal activity (SBA) and therefore, terminal complement blockage increases the risk of invasive disease by encapsulated bacteria (42–45). This has led to the concept that compared to C5 inhibition, specific inhibition of the AP may reduce the infection risk.

**Figure 1:**
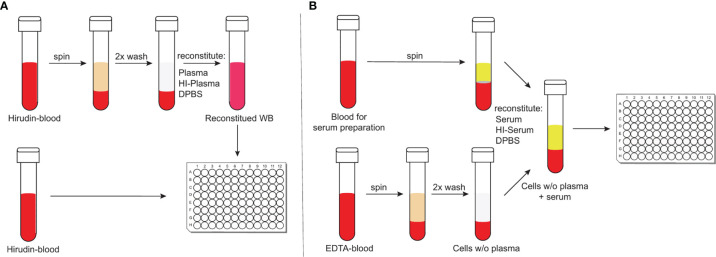
Schematic representation of the two physiological opsonophagocytosis assays. Hirudin-anticoagulated whole blood allows the assessment of bacterial killing in unprocessed whole blood or in reconstituted whole blood with either active or HI-plasma for internal controls **(A)**. DPBS-washed cells from EDTA-anticoagulated blood can be reconstituted with active serum and represents another physiological opsonophagocytosis assay that can be used to analyze previously stored serum samples **(B)**.

To study complement-mediated opsonisation, phagocytosis and killing of encapsulated bacteria, an assay that incorporates active complement and living phagocytes is needed. Widely-used opsonophagocytosis assays are typically based on isolated phagocytes or cell lines, such as HL-60 cells and exogenous standardized complement sources, such as baby rabbit complement (46, 47). Focusing on isolated cell types, however, reduces the complexity found within whole blood and affects the biological properties of the investigated cell types (48). Recently, more physiological methods to study complement-mediated opsonophagocytosis of bacteria have been reported that circumvent these limitations (49, 50). Hirudin-anticoagulated whole blood – by irreversible direct thrombin inhibition (51) – can be directly deployed for opsonophagocytosis assays after venous puncture. It, therefore, represents a particularly physiological assay with minimal effects on complement activity (**Figure 1A**) (49). Hirudin is used to replace lepirudin (52, 53), which has been withdrawn from the market. Another physiological assay makes use of cells from ethylenediamine tetraacetic acid (EDTA)-anticoagulated whole blood, which are washed and reconstituted with active serum and Ca^2+^ and Mg^2+^ to override the effect of chelating bivalent cations by EDTA (50), which interferes with complement activation (**Figure 1B**) (49). Here we compared results obtained with these two physiological opsonophagocytosis assays and investigated the relative contribution of opsonisation by antibodies, classical, lectin, alternative and terminal complement pathways to pneumococcal opsonophagocytosis. Pneumococcal killing was measured in the presence of inhibitors against complement fB, fD, C3, C5 or the mannose-binding lectin–associated protease 2 (MASP-2).

## 2 Methods

### 2.1 Ethical Approval

All experimental steps involving human specimens were approved by the Ethical Committee of Northwest and Central Switzerland (Ethikkommission Nordwest- und Zentralschweiz (EKNZ), Studie 2018-02341).

### 2.2 Measurement of Anti-Pneumococcal Capsule Antibody in Serum

Pneumococcal serotype-specific IgG concentrations were analyzed by the Vaccine Evaluation Unit (VEU) of Public Health England (PHE) in Manchester, UK, using the 007sp 12-plex pneumococcal IgG Bioplex assay. The lower limit of quantification (LLOQ) for all serotypes in the Pneumococcal IgG Bioplex assay was 0.10 µg/mL.

### 2.3 Bacterial Growth

A clinical isolate of *Streptococcus pneumoniae* (serotype 4, P1541) was used to assess complement-mediated opsonisation and phagocytic killing with the two blood assays. The pneumococcal strain was grown in 10 mL brain heart infusion broth (BHI, BD, Switzerland) for 7 h at 37°C. Subsequently, the culture was diluted 10-fold in fresh pre-warmed BHI broth and grown for 2 h at 37°C with 5% CO_2_ to logarithmic growth phase (OD_600_ ≈ 0.65). For opsonophagocytosis experiments, pneumococci were diluted in Dulbecco’s Phosphate-Buffered Saline containing MgCl_2_ and CaCl_2_ (DPBS, Sigma-Aldrich, Switzerland) to reach 8.4 ± 2.7 x 10^3^ CFU/mL in hirudin assays or 2.8 ± 1.0 x 10^3^ CFU/mL in ethylenediamine tetraacetic acid (EDTA) assays. CFUs were determined by serial dilution and plating on Columbia agar with 5% sheep blood (CSBA, BioMérieux, Switzerland). *Neisseria meningitidis* serogroup W case isolate 1682 (54) was grown in Frantz medium, as previously described (55). For opsonophagocytosis experiments, meningococci were diluted in DPBS to reach 3.5 ± 0.8 x 10^3^ CFU/mL in DPBS before adding them to blood.

### 2.4 Opsonophagocytic Killing Assays

#### 2.4.1 Blood Sampling

Study participants (age range 25-67, **Table 1**) had either been vaccinated previously with a 13-valent pneumococcal conjugate vaccine (PCV13, Prevenar 13, Pfizer; n=5) or were pneumococcal vaccine-naïve (n=4). Within this study, two physiological opsonophagocytic killing assays were compared using venous blood specimens from these nine healthy volunteers. The assay using hirudin-anticoagulated whole blood collected in S-monovette^®^ 1.6 mL tubes (Sarstedt, Germany) was compared to the assay using EDTA-anticoagulated whole blood collected in BD Vacutainer^®^ 4 mL tubes (BD, Switzerland) washed with DPBS and reconstituted with serum collected in Vacuette^®^ CAT serum tubes (Greiner Bio-One, Switzerland). Blood from an additional volunteer, who had recently received a MenACWY vaccine booster immunization was used for control experiments analyzing meningococcal clearance.

**Table 1:** Description of study participants with time since receiving the 13-valent pneumococcal conjugate vaccine (PCV13).

Subject no.	Age	Sex	Time since vaccination
**1**	32	Male	Naïve
**2**	43	Female	Naïve
**3**	25	Female	Naïve
**4**	25	Female	Naïve
5*	67	Male	4 months
**6**	25	Female	2 weeks
**7**	40	Male	6.5 years
**8**	29	Female	3.5 years
**9**	29	Male	3 years

*From this subject a serum sample from before pneumococcal vaccination was available and included for the analysis of anti-pneumococcal capsule antibody concentrations and opsonophagocytic titer analysis.

#### 2.4.2 Hirudin Method

For the hirudin assay, 80 µL hirudin-anticoagulated whole blood was added per well in a 96-well flat bottom culture microplate (Falcon™, ThermoFisher Scientific, Switzerland) and 10 µL of complement inhibitors or DPBS were added prior to inoculation with 10 µL bacteria. For intrinsic controls, hirudin-anticoagulated whole blood was centrifuged at 1’000 x g for 10 minutes at 4°C, plasma was removed and a part of it was heat-inactivated (HI) at 56°C for 30 minutes. The cell fraction was washed with ice-cold DPBS and reconstituted with the previously determined amount of either active plasma or HI-plasma. From all intrinsic controls, 90 µL were added per well to the 96-well plate (**Figure 1A**).

#### 2.4.3 EDTA Method

For the EDTA assay, serum collection tubes were kept at room temperature for 30 minutes to allow clotting. Tubes were spun at 1’700 x g at 4°C for 10 minutes and serum was transferred to 1.5 mL screw-cap microcentrifuge tubes. Tubes were either kept on ice, or stored at -80°C, while a fraction was heat-inactivated at 56°C for 30 minutes. EDTA-anticoagulated whole blood was centrifuged at 1’000 x g for 10 minutes at 4°C, washed twice with DPBS and reconstituted with the previously determined amount of either serum or HI-serum. As for the hirudin-assay, 80 µL of reconstituted blood and 10 µL of complement inhibitors or DPBS were added to a 96-well plate before inoculating with 10 µL of desired bacterial dilution. From all intrinsic controls, 90 µL were added per well to the 96-well plate. To analyze the effect of blood cells on bacterial killing without serum or plasma in both assays, cells were reconstituted with DPBS (**Figure 1B**).

#### 2.4.4 Inhibitors

To investigate the contribution of different complement components to the killing of *S. pneumoniae* and *N. meningitidis* in the two blood assays, selected complement inhibitors of the alternative, lectin and terminal pathway were used. Inhibitors of factor B [Iptacopan, LNP023, Novartis (56)], factor D [CMS487, Novartis (56)], C3 (CP-40, Bachem) as well as an anti-C5 antibody [LFG316, tesidolumab, Novartis (57, 58)] and an anti-MASP-2 antibody [narsoplimab (59, 60)] were diluted in DPBS, added to the blood samples and incubated for 5 minutes at room temperature before adding the bacteria. Inhibitor concentrations tested for LNP023 and CMS487 were 1 and 10 µM, for C3, C5 and MASP-2 inhibitors 1 and 50 µg/mL.

#### 2.4.5 Bacterial Clearance

After bacteria were added, the 96-well plates were incubated at 37°C with 5% CO_2_ and bacterial survival was analyzed by repeated sampling of 10 µL after 60, 180 and 300 minutes and plating on CSBA plates. Agar plates were subsequently incubated overnight at 37°C with 5% CO_2_, colonies were counted manually, and data converted into CFU per mL.

#### 2.4.6 Opsonophagocytic Titer Determination

Opsonophagocytic titer were determined using the EDTA-based whole blood assay described above, but with modifications. In short, after 4-fold serial dilution of heat-inactivated sera in a 96-well flat bottom culture microplate leaving 25 µL diluted serum per well, 45 µL washed blood cells from a vaccine-naïve donor were added before adding 10 µL of diluted bacteria (as described above) and 20 µL of baby rabbit complement (Cedarlane). The 96-wells plates were incubated at 37°C with 5% CO_2_ on a rotator. Bacterial killing was analyzed at 60 minutes post inoculation. Opsonophagocytic titers represent the reciprocal of the serum dilution with >50% killing compared with bacterial growth in the complement control wells. Serum samples with undetectable titers, representing a titer lower than our lowest dilution (<4), obtained the value 2.

### 2.5 Longitudinal Study With Paired Pre and Post Vaccination Samples

Eleven healthy volunteers were recruited upon informed and written consent. Two subjects had previously been vaccinated with pneumococcal vaccines (**Table 2**). Eight subjects received a single dose of a 13-valent conjugate pneumococcal vaccine (Prevenar 13, PCV13, Pfizer) and three subjects received a single dose of a 23-valent unconjugated pneumococcal polysaccharide vaccine (Pneumovax 23, PPV23, MSD). Serum and blood from venous blood were taken before, 2 weeks and 2 months after vaccination. Pneumococcal serotype-specific IgG concentrations were analyzed as described above. Opsonophagocytic killing was assessed using the hirudin assays described above inoculated with a bacterial dilution containing 7.3 ± 3.1 x 10^3^ CFU/mL.

**Table 2:** Description of study participants including previously administered pneumococcal vaccines.

Subject no.	Age	Sex	Previous pneumococcal vaccines	Pneumococcal vaccine received during study
**10**	32	Female	PPV23^*^ (2012)	PCV13^**^
**11**	38	Female	PPV23 (2007); PCV13 (2013)	PCV13
**12**	33	Male	-	PCV13
**13**	25	Female	–	PCV13
**14**	31	Male	-	PCV13
**15**	35	Female	–	PCV13
**16**	26	Male	-	PCV13
**17**	25	Male	–	PCV13
**18**	31	Female	-	PPV23
**19**	45	Male	–	PPV23
**20**	31	female	-	PPV23

*23-valent pneumococcal polysaccharide vaccine.

**13-valent pneumococcal conjugate vaccine.

### 2.6 Statistical Analysis

Differences of anti-pneumococcal immunoglobulin IgG between vaccine-naïve and previously vaccinated study participants were assessed using a two-tailed unpaired t test for parametric data on GraphPad Prism (Prism 8; GraphPad Software Inc., San Diego, USA). Difference in opsonophagocytic titers were assessed using a non-parametric Mann-Whitney test. Spearman’s non-parametric correlations was used to correlate the IgG levels with the opsonophagocytic titers. Pearson’s correlation was used to correlate serum anti-pneumococcal immunoglobulin IgG levels with the log-transformed numbers of surviving bacteria in presence of complement inhibitors. P values of ≤0.05 were considered to be statistically significant.

## 3 Results

### 3.1 Induction of Capsule Polysaccharide-Specific Antibodies by PCV-13 Vaccination

**Figure 2:**
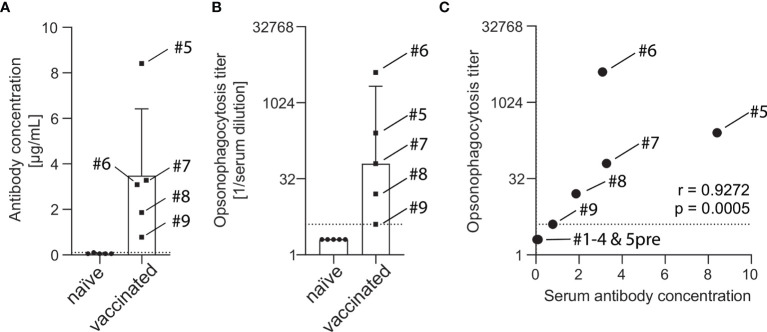
Concentration of capsule polysaccharide-specific IgG and opsonophagocytic titers in sera of vaccine-naïve and PCV13-vaccinated subjects. A pneumococcal IgG Bioplex assay was used to measure antibody concentrations in the serum of five vaccine-naïve and five PCV13-vaccinated subjects. Vaccinated study participants had statistically significant higher antibody concentrations than the vaccine-naive group (3.48 ± 2.93 µg/mL vs. 0.06 ± 0.02 µg/mL, p = 0.031). The box indicates the sample mean and the standard deviation and the dotted line represent the lower limit of quantification **(A)**. Pneumococcal opsonophagocytic titer were determined using PBS-washed EDTA-anticoagulated blood cells reconstituted with diluted heat-inactivated serum and 20% active baby rabbit complement. Vaccinated study participants had statistically significant higher titers than the vaccine-naïve group (median titer 64 vs. 2, p = 0.0079). Boxes indicate sample median and interquartile range and the dotted line represents the lowest quantifiable opsonophagocytic titer **(B)**. IgG levels and opsonophagocytic titers revealed a strong positive correlation **(C)**. Serum from subject #5 before vaccination was available for these analyses.

Serum of vaccine-naïve and PCV13-vaccinated subjects were analyzed for serotype 4 capsule polysaccharide-specific IgG. While sera of all five vaccine-naïve subjects showed IgG concentration below or at the lower limit of quantification (0.10 µg/mL), sera of five PCV13-vaccinated study participants contained between 0.78 and 8.41 µg/mL capsule polysaccharide-specific antibodies (**Figure 2A**). Detectable opsonophagocytic titers were only found in vaccinated subjects and ranged from 4 to 4096 with a median titer of 64 (**Figure 2B**). Opsonophagocytic titers and polysaccharide-specific IgG concentrations demonstrated a significant positive correlation (Spearman’s r = 0.927, p < 0.001, **Figure 2C**).

### 3.2 Role of Antibodies and Complement Activation During Pneumococcal Opsonophagocytosis

**Figure 3:**
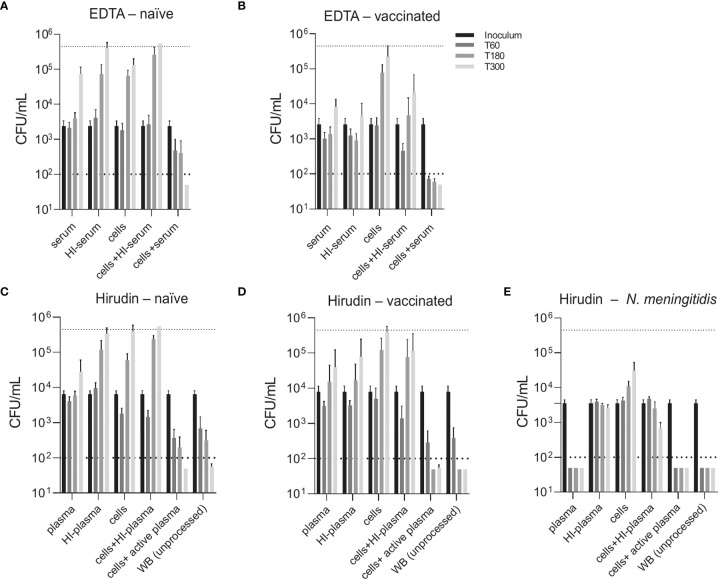
Contribution of active complement and antibodies to opsonophagocytic killing of pneumococci. Pneumococcal killing was assessed in either an assay based on DPBS-washed cells from EDTA-anticoagulated blood reconstituted with active or HI serum [EDTA assay; **(A, B)**] or in a hirudin-anticoagulated whole blood (WB) assay [Hirudin assay; **(C, D)**] comparing vaccine-naïve **(A, C)** and PCV13-vaccinated individuals **(B, D)**. Cumulated results for all individuals per group are shown. Meningococcal killing in hirudin-anticoagulated whole blood of a MenACWY-vaccinated volunteer **(E)** served as a control for direct killing by the MAC. Bars indicate CFU/mL at start of inoculation and 60, 180 and 300 minutes after inoculation. Dashed lines demonstrate the lower detection limit ( < 100 CFU/mL) and the upper limit ( > 500’000 CFU/mL) for exact quantification. WB, whole blood; HI, heat-inactivated.

**Figure 4:**
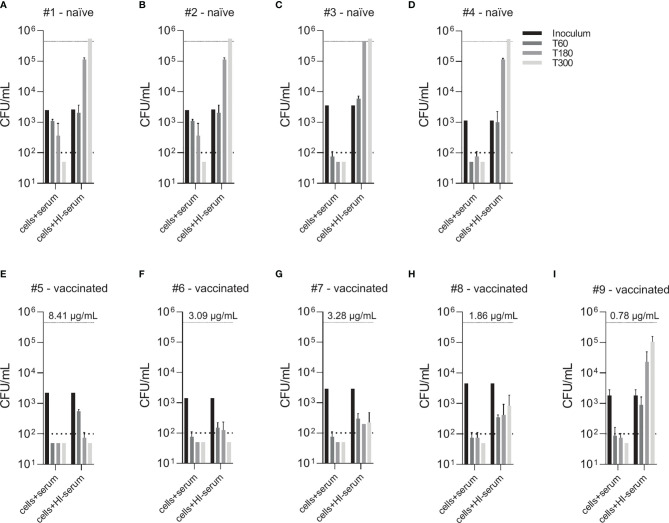
Effect of vaccine-induced anti-pneumococcal capsule antibodies in the absence of active complement. Pneumococcal killing was assessed in an opsonophagocytosis assay based on DPBS-washed cells from EDTA-anticoagulated blood reconstituted with active or HI serum, comparing individual vaccine-naïve **(A–D)** and PCV13-vaccinated individuals **(E–I)**. Concentration of serotype 4 capsule polysaccharide-specific IgG in sera of vaccinated subjects is displayed. Bars indicate CFU/mL at time of bacterial inoculation and after 60, 180 and 300 minutes. Dashed lines demonstrate the lower detection limit ( < 100 CFU/mL) and the upper limit ( > 500’000 CFU/mL) for exact quantification.

To compare two physiological opsonophagocytosis assays based on either DPBS-washed cells from EDTA-anticoagulated blood reconstituted with active serum or hirudin-anticoagulated whole blood, we assessed bacterial killing in blood samples of nine healthy volunteers. Of these, four subjects were pneumococcal vaccine-naïve and five had received the 13-valent pneumococcal conjugate vaccine (PCV13). Both assays produced highly comparable results and demonstrated that active complement and/or high titers of capsule specific antibodies are required for phagocytic killing of pneumococci (**Figure 3**). Serum/plasma alone, blood cells alone and cells reconstituted with heat-inactivated serum/plasma did not cause bacterial killing, but rather allowed pneumococcal proliferation over the assay duration of 5 hours. Unprocessed whole blood from both PCV13-vaccinated and vaccine-naïve subjects or blood cells reconstituted with the corresponding active serum killed the pneumococci within 5 hours after inoculation in both assays (**Figures 3A–D** and **4**). Pneumococcal killing might occur faster in samples from PCV13-vaccinated subjects, where a decreased colony count was detectable as early as one hour after inoculation. Notably, however, two vaccine-naïve subjects also showed complete killing within one hour after inoculation (**Figures 4C, D**). Pneumococcal proliferation in blood cell samples reconstituted with heat-inactivated serum/plasma from vaccinated, but not from vaccine-naïve subjects, was slightly reduced (**Figures 3A–D**).

Generally, the two assay formats yielded very similar results and highlighted the importance of active complement and/or antibodies for efficient opsonophagocytic killing of pneumococci. In contrast to these findings, a control experiment confirmed that, as expected, anti-capsular antibodies and active complement are sufficient for effective killing of the Gram-negative bacterium *N. meningitidis* in the absence of phagocytes (**Figure 3E**).

Comparison of individual subject data revealed that bacterial killing was also observed with blood cell samples reconstituted with heat-inactivated serum of vaccinated study participants, if the concentration of vaccine-induced anti-pneumococcal capsule antibodies was high (**Figure 4**). In particular, sera from subjects #5, 6 and 7 (**Figures 4E–G**), which contained capsule specific antibody concentrations >2 µg/mL, representing opsonophagocytic titers ≥64 (**Figure 2**), showed pneumococcal killing even in the absence of active complement, indicative for antibody-dependent, but complement-independent pneumococcal killing.

### 3.3 In Vaccine-Naïve Subjects, Pneumococcal Opsonophagocytosis Is Dependent on Alternative Pathway Activation

**Figure 5:**
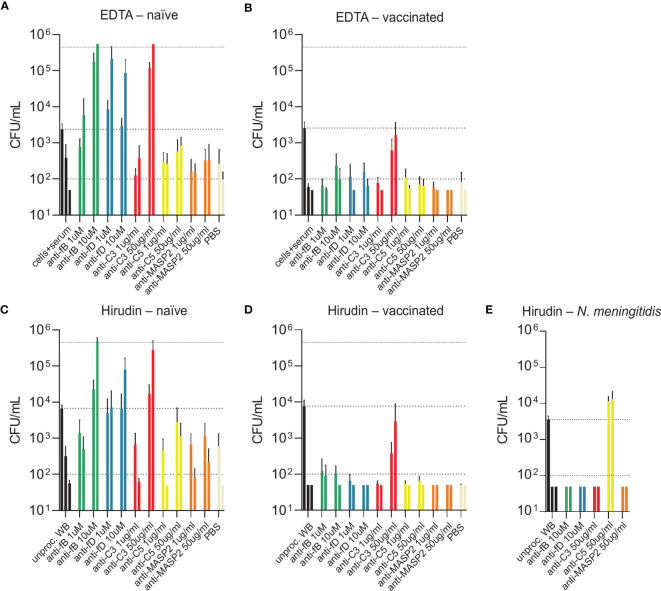
Bacterial opsonophagocytosis in the presence of complement inhibitors. Effects of different complement inhibitors on pneumococcal killing were assessed in parallel with either DPBS-washed cells from EDTA-anticoagulated blood reconstituted with active serum **(A, B)** or with hirudin-anticoagulated whole blood **(C, D)**. Cumulated results for three individuals per group are shown. Meningococcal killing in the presence of complement inhibitors in hirudin-anticoagulated whole blood of a MenACWY-vaccinated volunteer **(E)** served as a control. Bars indicate bacterial CFU/mL at 3 and 5 h after inoculation (respectively, left and right bar for each condition). In the control conditions (black), the inoculum titer is depicted as the first bar and further indicated as dashed line. Other dashed lines demonstrate the lower detection limit and the upper limit for bacterial quantification. WB, whole blood.

**Figure 6:**
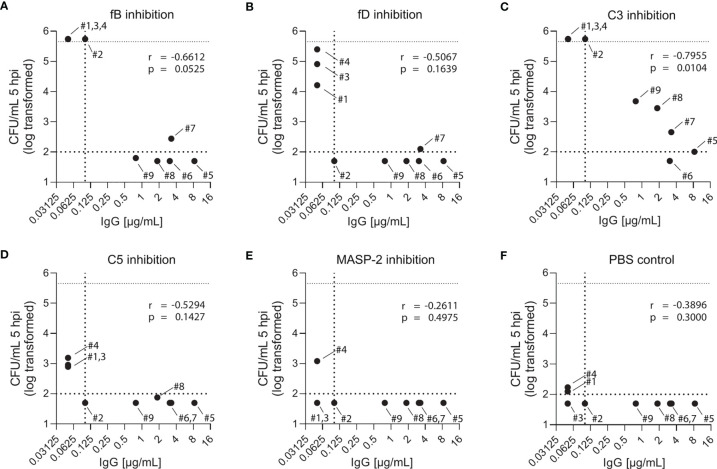
Effect of complement inhibition and anti-pneumococcal capsule antibody concentrations on pneumococcal killing in individual study participants. Pneumococcal killing was assessed in an opsonophagocytosis assay based on DPBS-washed cells from EDTA-anticoagulated blood reconstituted with active serum comparing individual vaccine-naïve and PCV13-vaccinated subjects in the presence of 10µM fB **(A)** or fD **(B)** inhibitor, 50 µg/mL C3 **(C)**, C5 **(D)** or MASP-2 **(E)** inhibitor and PBS control **(F)**. Log-transformed bacterial number in CFU/mL five hours after bacterial inoculation were correlated with capsule-specific IgG concentrations in serum of each individual study participant. Dashed horizontal lines demonstrate the lower detection limit and the upper limit for bacterial quantification, while the dashed vertical line represents lower limit for IgG concentration quantification. hpi: hours post inoculation.

While blood from both PCV13-vaccinated and vaccine-naïve subjects induced pneumococcal killing after five hours of incubation (**Figures 3**, **4**), striking differences were observed in the presence of AP inhibitors. In vaccine-naïve subjects, pneumococcal killing was completely abrogated when fB (with 10 µM LNP023) or fD (with either 1 or 10 µM CMS487) was inhibited (**Figures 5A, C**). In contrast, AP inhibition caused only a slight delay in killing at 3 hours and had no effect at 5 hours when blood samples from subjects who had previously been vaccinated with PCV13 were analyzed (**Figures 5B, D**). Correlation of anti-capsule antibody concentrations and pneumococcal clearance in presence of AP inhibition showed that all vaccinated subjects, independent of the anti-capsule antibody concentration, killed the pneumococci completely within 5 hours after bacterial inoculation (**Figures 6A, B**). Notably, complete pneumococcal clearance in presence of fD inhibitor was also observed in the non-immune subject #2 (**Figure 6C**), which was the only vaccine-naïve subject with detectable anti-capsule antibody levels (0.1 µg/mL).

Inhibition of C3 with CP-40 at a concentration of 50 µg/mL completely prevented pneumococcal killing in samples of vaccine-naïve participants (**Figures 5A, C**). In contrast, in samples of vaccinated individuals the effect depended on the concentration of the capsule-specific antibodies. While the cumulated results showed an overall impact of C3 inhibition (**Figures 5B, D**), detailed analyses of individual subjects provided evidence for complement-independent opsonophagocytic killing in participants with high concentrations of capsule-specific antibodies (**Figure 6C**). In reconstituted blood samples from subjects #5, 6 and 7, which contained antibody concentrations >2 µg/mL (**Figure 2**), efficient killing was observed when C3 was blocked (**Figure 6C**). In samples of the vaccinated subjects #8 and 9 with low antibody concentrations, bacterial proliferation was still contained, whereas inhibition of C3 provoked a massive proliferation of pneumococci in vaccine-naïve subjects (#1,2,3 and 4). A significant inverse correlation between serum IgG concentration and the number of surviving pneumococci 5 hours after inoculation was observed (**Figure 6C**), indicating that high levels of capsule-specific IgG are triggering pneumococcal opsonophagocytosis even in the absence of C3. Results of C3 inhibition were similar to obtained results with heat-inactivated sera (**Figure 4**).

Irrespective of the vaccination status, inhibition of MASP-2 or C5 did not substantially impair bacterial killing, except for a marginal effect of C5 in vaccine-naïve subjects (**Figures 5A–D** and **Figures 6D, E**). In control experiments, inhibition of C5 completely blocked killing of *N. meningitidis* with serum from a MenACWY-vaccinated individual (**Figure 5E**).

### 3.4 The Effect of Pneumococcal Vaccinations on Complement-Dependent Opsonophagocytosis

**Figure 7:**
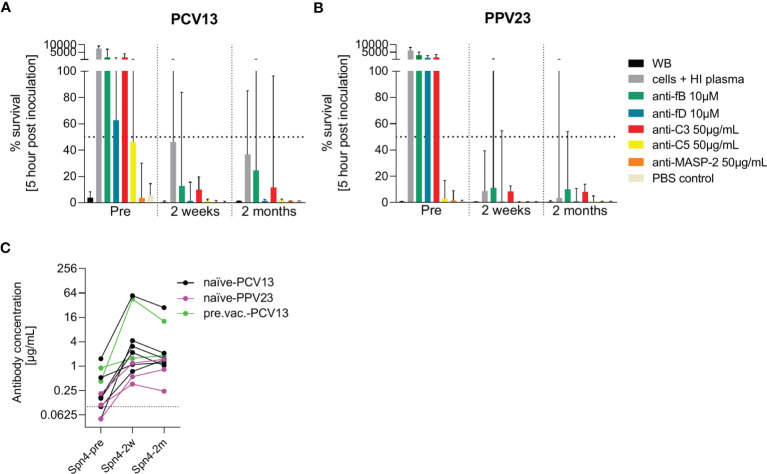
Effect of PCV13 and PPV23 vaccination on bacterial opsonophagocytosis in the presence of complement inhibitors. The effect of pneumococcal vaccination on complement-dependent opsonophagocytosis was assessed in hirudin-anticoagulated whole blood. Cumulated results for eight PCV-13 vaccinated subjects **(A)** and three PPV23 vaccinated participants **(B)** are shown. Bars indicate the median percentage of surviving bacteria five hours after inoculation. The dashed line represents 50% killing compared to the inoculum titer. Antibody concentrations in the serum of all study participants were measured before, two weeks and two months after vaccination by a pneumococcal IgG bioplex assays **(C)**. The dashed line in **(C)** represents the lower limit of quantification. PCV13: 13-valent pneumococcal conjugate vaccine; PPV23: 23-valent pneumococcal polysaccharide vaccine; WB: whole blood, HI: heat-inactivated.

Eleven additional study participants were recruited and vaccinated with either PCV13 or PPV23 (**Figures 7A, B**). Pneumococcal opsonophagocytosis in the presence or absence of complement inhibitors was assessed before as well as two weeks and two months post vaccination. There was an increase in capsule polysaccharide-specific IgG concentrations upon vaccination in all eleven study participants (**Figure 7C**). Inhibition of C3 or the alternative pathway abrogated pneumococcal opsonophagocytosis in whole blood. The effect of fD inhibition was slightly less prominent in the PCV13 group, potentially due to pre-existing immunity in some subjects. Two weeks after vaccination, pneumococcal opsonophagocytic killing in whole blood was not prevented by inhibition of the alternative complement pathway. While inhibition of fD did not prevent complete pneumococcal killing, inhibition of fB slowed down pneumococcal killing slightly. Also C3 inhibition resulted in incomplete pneumococcal killing. Inhibition of C5 or MASP-2 did not affected pneumococcal killing. Opsonophagocytosis patterns observed with whole blood collected two weeks and two months after vaccinations were similar. Vaccination with PCV13 and PPV23 had similar effects.

## 4. Discussion

Although there is general agreement that both complement and anti-pneumococcal antibodies contribute to opsonophagocytosis-mediated immune defense against *S. pneumoniae* (11, 61–63), only limited data are available on the relative contribution of complement- and direct antibody-mediated opsonisation. To address this, we compared two physiologic opsonophagocytosis assays adapted from recently published methods. A hirudin-anticoagulated whole blood assay (49) was directly compared to an assay based on cells from EDTA-anticoagulated blood, where plasma was removed, cells were washed and supplemented with active serum (50). The two assays yielded highly comparable results, with both assays demonstrating the relative importance of antibodies and of AP and CP activation for opsonophagocytic killing of pneumococci. A major advantage of the hirudin assay is that minimal processing results in highly physiologic conditions, furthermore representing an easy-to-use protocol. On the other hand, the serum-reconstituted EDTA assay allows analyzing the effect of different serum samples from different time points repeatedly and in direct comparison with the same cell preparation. However, this assay involves processing steps, which might affect the biology of investigated cell types and also affect complement activation through coagulation (48, 64). Depending on the scientific question addressed, one or the other assay might be more favorable to use.

Both assays showed that AP activation plays a key role in opsonophagocytosis in vaccine-naïve subjects, whereas inhibition of fB and fD did not affect pneumococcal killing in PCV13-vaccinated individuals, independent of anti-pneumococcal capsule antibody concentration and opsonophagocytic titer (**Figures 6A, B**). Even low anti-pneumococcal capsule antibody concentrations were sufficient to clear pneumococci from blood *via* antibody-mediated CP and/or Fc receptor-mediated phagocytosis. Opsonophagocytosis data with paired blood samples from study participants before and after vaccination confirmed these conclusions (**Figures 7A, B**).

Experiments with both heat-inactivated and C3-inhibited sera showed that in the absence of active complement, but in presence of high anti-capsule IgG serum concentrations, representing high opsonophagocytic titers, opsonophagocytosis is still efficient, which is indicative for complement-independent Fc receptor-mediated phagocytosis. Therapeutic inhibition of C3, however, might leave some vaccinated subjects with increased risk of acquiring pneumococcal infections, when vaccine-induced antibody levels are low (**Figure 6C**).

C5 cleavage, which induces MAC formation and release of the pro-inflammatory C5a anaphylatoxin is crucial for meningococcal clearance from whole blood (52). Control experiments with *N. meningitidis* confirmed this importance and showed that inhibition of C5 blocked meningococcal clearance in whole blood (**Figure 5E**), as previously reported (1–5, 52). Nevertheless, inhibiting C5 during experimental pneumococcal infection did not affect bacterial killing in vaccinated individuals and only marginally affected it in vaccine-naïve subjects. Furthermore, our data indicate that the lectin pathway may not be essential for immune defense against pneumococci even in vaccine-naïve subjects, all of whom presented some degree of natural immunity. This is in line with observational studies showing no association between LP deficiencies and the risk for acquiring pneumococcal infections (22, 23).

Limitations of this study include a relatively small initial sample size. However, main conclusions were fully verified by testing paired blood samples collected from subjects both before and after vaccination. Moreover, we focused on one pneumococcal serotype as blood volumes approved by the ethics committee did not allow to perform whole-blood assays with our panel of complement inhibitors for multiple serotypes.

Taken together, our results show that fB and fD inhibitors, which still allow complement activation through antibody-mediated CP, do not abrogate pneumococcal opsonophagocytosis in PCV13- and PPV23-vaccinated individuals. Hence, AP inhibitors can safely be developed in the clinic for potential treatments in patients with diseases involving the complement system. Treatment with AP inhibitors should be accompanied by vaccination with a pneumococcal vaccine to elicit anti-capsule antibodies and B cell memory responses that enable rapid humoral immune responses against pneumococcal infections.

## Data Availability Statement

The raw data supporting the conclusions of this article will be made available by the authors, without undue reservation.

## Ethics Statement

The studies involving human participants were reviewed and approved by Ethical Committee of Northwest and Central Switzerland (Ethikkommission Nordwest- und Zentralschweiz (EKNZ), Studie 2018-02341). The patients/participants provided their written informed consent to participate in this study.

## Author Contributions

LM and EI contributed equally to this study during study conceptualization, data acquisition, analysis and interpretation as well as drafting the manuscript. GP contributed to study conceptualization and design, data analysis and interpretation and revised the manuscript. AS, CT, NZ, TH, and MK contributed to study conceptualization and design and revised the manuscript. All authors contributed to the article and approved the submitted version.

## Funding

GP received funding to conduct the study under a research agreement contract between Novartis Pharma AG and the Swiss Tropical and Public Health Institute.

## Conflict of Interest

AS, CT, NZ, TH and MK are full-time employees of Novartis.

The remaining authors declare that the research was conducted in the absence of any commercial or financial relationships that could be construed as a potential conflict of interest.​

The authors declare that this study received funding from Novartis Pharma AG. The funders initiated study design and decision to implement opsonophagocytosis assays to compare the activity of the tested drugs. The funders had no role in data collection and analysis.

## Publisher’s Note

All claims expressed in this article are solely those of the authors and do not necessarily represent those of their affiliated organizations, or those of the publisher, the editors and the reviewers. Any product that may be evaluated in this article, or claim that may be made by its manufacturer, is not guaranteed or endorsed by the publisher.
